# Conservation outreach that acknowledges human contributions to climate change does not inhibit action by U.S. farmers: Evidence from a large randomized controlled trial embedded in a federal program on soil health

**DOI:** 10.1371/journal.pone.0253872

**Published:** 2021-07-01

**Authors:** Paul J. Ferraro, Jacob Fooks, Rich Iovanna, Maik Kecinski, Joel Larson, Ben S. Meiselman, Kent D. Messer, Mike Wilson

**Affiliations:** 1 Department of Environmental Health and Engineering, Whiting School of Engineering, Carey Business School, Johns Hopkins University, Baltimore, Maryland, United States of America; 2 New York Life Insurance Company, New York, New York, United States of America; 3 United States Department of Agriculture, Farm Service Agency, Washington, District of Columbia, United States of America; 4 Department of Applied Economics and Statistics, University of Delaware, Newark, Delaware, United States of America; 5 University of Minnesota, Minneapolis, Minnesota, United States of America; 6 United States Department of the Treasury, Office of Tax Policy, Washington, District of Columbia, United States of America; 7 United States Department of Agriculture, Natural Resource Conservation Service, Washington, District of Columbia, United States of America; Universidad de Murcia, SPAIN

## Abstract

Technologies and practices that reduce the environmental impacts of US agriculture are well documented. Less is known about how best to encourage their adoption. We report on the results of a large randomized controlled trial conducted with nearly 10,000 agricultural producers in the United States. The experiment was embedded in US Department of Agriculture outreach efforts to improve soil conservation practices. USDA varied the content of mailings to test two sets of competing theories about outreach to agricultural producers. Contrary to conventional wisdom, we find no evidence that acknowledging the link between climate change and agricultural production discourages conservation action. Furthermore, we find that producers who were invited to a webinar were less likely to take any action to learn more about conservation practices than producers who were not told about the webinar, a result that runs counter to the popular wisdom that offering more options leads to more action.

## Introduction

To reduce the negative effects of agricultural activities on soil health and other environmental conditions, scholars and practitioners have developed a wide range of conservation practices and policy targets. Known mitigation practices in agriculture are estimated to potentially reduce annual anthropogenic emissions by 4 GtCO2 eq/yr by 2030 [1]. Yet for these soil health conservation practices to realize their potential, agricultural producers must use them.

Promoting conservation practices in agricultural production is fundamentally about changing human behaviors [2]. Empirical evidence about how best to change human behaviors is often best supplied through large randomized experiments in real-world settings with observed, rather than hypothetical, human behaviors [3]. Such experiments, however, are rare in the conservation literature [4–8]. Partially due to this rarity, there are large gaps in our understanding about how to induce behavior change among agricultural producers and other actors who influence conservation outcomes [9].

We report results from a large-scale randomized field experiment embedded in a US Department of Agriculture (USDA) soil conservation outreach initiative. USDA sent a series of outreach mailings to nearly 10,000 agricultural producers to encourage alternative conservation farming practices. Each producer was randomly assigned to one of several treatment groups, and each treatment group received a different set of mailings. Because treatment was randomly assigned as part of an experiment, we can be precise about how confident we are that differences in the average behavior of the treatment groups are attributable to differences in the treatment mailings [10].

The experimental treatment conditions were motivated by two debates within USDA about how best to encourage agricultural producers to adopt climate-related soil conservation practices (four of the co-authors were USDA employees at the time of the experiment). The first debate concerned the potential impact of acknowledging agricultural producers’ contributions to climate change. A major motivation for outreach promoting soil conservation practices is to mitigate climate change by reducing carbon emissions, and it would be natural to say so in outreach to agricultural producers. However, based on field staff opinions and surveys in which producers stated that they do not believe humans contribute to climate change [11], it had become conventional wisdom at federal agencies in the United States, including at USDA, that acknowledging human contribution to climate change in outreach with US producers would be detrimental to efforts to change behavior [12]. Contrary to the conventional wisdom, we find no evidence that acknowledging human contribution to climate change was detrimental to conservation outreach.

The second debate in USDA concerned the impact of offering producers too many or too few options for action. USDA outreach typically offers producers a menu of possible actions, under the assumption that producers are more likely to act when they have more actions to choose from. Contrary to USDA’s typical approach, a contingent at USDA argued that producers are more likely to act when communication directs their attention towards a single option than when their attention is divided across several options. Consistent with the latter hypothesis, we find that producers were more likely to take an initial step towards learning about conservation practices when they were given one option (visit a website) than when they were given two options (visit a website or attend a webinar).

Our outcome measure is the website visitation rate generated by each treatment condition. Visiting a website to learn more about conservation practices is relatively low effort and early on the pathway to adopting those practices. Ideally, we would also want to examine subsequent adoption of the actual conservation practices as well. Unfortunately, our data sources do not permit us to observe the impact of the treatment on downstream adoption of conservation practices, so we focus on the intermediate outcome that we do observe.

Below, we provide additional background on soils farmed by the targeted agricultural producers, survey evidence about climate change beliefs of agricultural producers, and the literature on how adding options can affect decisions. We then elaborate on the experimental design, report behavioral impacts from the experimental treatments, and discuss the implications for outreach and research in behavior change for conservation.

## Background

### Histosol soils

The mailings sent by USDA were targeted to agricultural producers with farms on histosol soils. Histosol soils can be excellent for farming because they have high nutrient content and water-holding capacity. Yet when drained, tilled, limed, and cropped, they are also prone to oxidation and subsidence. These biophysical processes can harm the environment in a variety of ways, including by releasing a substantial amount of greenhouse gases into the atmosphere [13–16]. To reduce these emissions, producers could adopt alternative conservation farming practices like zero tillage or cover crops, but such practices are comparatively rare on productive histosol soils.

### Climate beliefs as an impediment to conservation outreach

Surveys suggest most US agricultural producers do not believe in anthropogenic climate change [11,17–19]. For instance, in a 2012 survey of 4,778 producers in the US Corn Belt, 66% reported believing climate change was occurring, but only 8% attributed it mostly to human behavior [11]. The researchers who conducted the Corn Belt survey concluded it was best to avoid acknowledging human contributions to climate change in conservation outreach to producers [11], other researchers have reached similar conclusions [20], and this view had become conventional wisdom at USDA.

However, people often behave in ways that are not consistent with their stated beliefs. American producers’ responses to a question about “belief in climate change” could say more about their cultural affinity than about their comprehension of climate science or their willingness to change behavior [21,22]. Climate change is a cultural flashpoint, and certain ways of asking about it may elicit affirmations of identity rather than truthful reports of beliefs [21,22]. However, if communication about climate change is designed to avoid threatening a producer’s identity, it may be possible to make a compelling case for mitigating carbon emissions despite producers’ surveyed beliefs. Indeed, in a less discussed result from the same Corn Belt survey [11], only 33% of producers disagreed with the statement, “I should reduce greenhouse gas emissions from my farm operation.” Thus, producers may be open to reducing their own carbon emissions even if they do not publicly acknowledge that climate change is mostly attributable to human behavior.

### How adding options can affect decisions

Classical economic models of human behavior suggest that it never hurts to have more options. A person evaluates the options available to her and chooses the option that is best according to her preferences. If an additional option becomes available, the person would either choose it if it is better or stick with her previous choice if the new option is worse. One formalization of this concept is called the weak axiom of revealed preference [23].

However, a large body of empirical evidence, supported by behavioral modeling, has identified several phenomena that run contrary to the classical logic. In one phenomenon called the decoy effect, adding a decoy option that is clearly inferior to one of the existing options has been shown to be capable of changing a person’s decision [24]. In another phenomenon called choice overload, adding options can interfere with the process of making decisions, particularly when the person making the decision is unsure about how to evaluate and value complex alternatives [25].

Because most USDA programs are voluntary, producers who interact with USDA generally have the option to do nothing, plus whatever options USDA makes salient. USDA often applies classical logic to conservation outreach—offering a menu of options under the assumption that more options could not hurt and may lead to more action. But as demonstrated by phenomena documented in the literature, such as the decoy effect and choice overload, adding an option could lead more producers to choose the option of doing nothing.

## Materials and methods

### Sample

USDA sent mailings to all agricultural producers who met the following selection criteria: (1) present in USDA’s Service Center Information Management System database, which tracks all interactions with the USDA Farm Service Agency, (2) cultivated crop land on at least one field where 20% of total field acreage was histosol soils, according to the USDA Natural Resources Conservation Service’s Soil Survey Geographic Database, (3) located in one of the seven states in which histosol soils are common and similar cropping patterns are used (Iowa, Illinois, Indiana, Michigan, Minnesota, Ohio, and Wisconsin), and (4) had at least $100,000 in annual revenue. There were 9,855 agricultural producers who met the selection criteria and comprise the experimental sample.

The purpose of the mailings was to get producers started along a path that would eventually lead to adopting conservation practices. The theory of change in USDA conservation outreach programs is that the first step towards adopting practices is to learn basic information about the conservation practices and related assistance programs. These initial steps of learning about practices and the related programs of financial and technical assistance lead to the step of adopting the practices.

### Experimental treatments

Each producer in the sample was sent three mailings—a letter on 15 March 2016, followed by a reminder postcard on 21 or 22 March 2016 and a second reminder postcard on 10 May 2016 (S1–S3 Figs). All letters explained that histosol soils are fragile and farming them can harm the environment. The letters invited recipients to visit a website to learn more about histosol soils, conservation practices, and USDA programs that support those practices.

Producers were randomly assigned to a treatment status. All outreach mailings emphasized the private benefits of the practices, framed as “soil health.” However, the description of the public benefits was experimentally varied, and the number of options for action was experimentally varied. As discussed earlier, these treatments were motivated by debates within USDA about which approaches to conservation outreach would be most successful.

The description of public benefits was varied to test whether acknowledging the link between greenhouse gas emissions and climate change impedes outreach. As shown in Table 1, half of the producers in the sample were assigned to the *climate treatment group*, and half to the *climate control group*. As shown in the S1–S3 Figs, agricultural producers assigned to the climate treatment group were told that one consequence of soil breakdown is the release of greenhouse gases that contribute to climate change. In contrast, mailings to the climate control group did not mention greenhouse gas emissions or climate change. Instead, control mailings framed the conservation practices in terms of preserving local water quality, which is a common theme of USDA outreach with producers and unlikely to activate the recipient’s cultural identity.

**Table 1:** Treatment assignment.

			Climate treatments	
			Acknowledge anthropogenic climate change	No mention of climate change
			50%	50%
**Option treatments**	**One option: Website only**	33%	1629	1629
	**Two options: Website and webinar**	67%	3263	3263

This is a 2x2 experimental design in the sense that assignment to one of the two “climate” treatments was statistically independent from assignment to one of the two “option” treatments. Concurrently with the experiment, 71 producers excluded from the above table received a phone call encouraging webinar attendance. Given that the phone call was a substantially different treatment than the mailings, we do not present an analysis of the effect of the phone call on behavior.

The number of options for initial action was varied to test whether agricultural producers are more likely to seek out information about conservation programs if they are offered two options for taking steps to learn about conservation practices or just one. One-third of the producers in the sample were invited only to visit a website, and two-thirds of the producers were invited both to visit a website and to attend a USDA webinar on 31 March 2016. Two-thirds of the sample, rather than half, were offered the webinar because some USDA extension staff, believing that offering more options was best, wished to offer the session to as many producers as requirements for statistical power would allow. The webinar was an hour-long online session run by USDA extension experts where producers could ask questions and learn in greater depth about the practices and programs. (The recorded session can be viewed at https://goo.gl/KN82vG.)

The comparison between producers who were invited to the website only (one option) and producers who were invited to the website and webinar (two options) is a test of the impact that adding an additional option has on a decision. Although the experiment does not provide a good test of choice overload, which the literature defines as involving larger choice sets with more complex choices, it is nevertheless true that the experimental design was motivated by a debate at USDA that followed the logic of choice overload. Thus, we take note of choice overload as an inspiration for the option treatments but regard the experiment as shedding light on the less specific question of how adding options can affect decisions.

### Measurement of behavioral outcomes

The primary outcome is webpage visitation rates. Mailings for the treatment conditions included distinct links to websites with the same content. Visits to the pages of these websites were tracked separately for each treatment condition, so the treatment that produced the visit to the specific pages of the website is known. However, the identity of the visitor is not known. Care was given to ensure that these websites could only be accessed through direct connection to this experiment design, and thus represented agricultural producers in the study. Webpage visitation rates are a measure of total actions taken because farmers registered for the webinar through the website.

Our primary analyses assess (1) the difference in webpage visitation rates between the climate treatment group and the climate control group and (2) the difference in webpage visitation rates between the multiple-option treatment group and the single-option control group. Formally, we test the two-sided null hypothesis of no effect of the climate treatment relative to the climate control; and the two-sided null hypothesis of no effect of the multiple-option treatment relative to the single-option control.

### Estimators and confidence intervals

Webpage visit rates are constructed by dividing the total number of visits to the specified pages by the number of producers sent mailings in that treatment arm. To construct confidence intervals, it is assumed that the number of website visits for a particular invitation is an independent draw from a Poisson distribution, which has the characteristic that the mean is equal to the variance. We apply critical values from the t-distribution when constructing confidence intervals because, under the assumption that of independent draws, the Central Limit Theorem applies to the sample mean visit rate.

The same procedure is used for constructing all the confidence intervals related to website visits per invitation. To illustrate, we describe an example calculation: The upper bound for the 95% confidence interval for homepage visits per climate change invitation is constructed by adding the two-tailed critical value (1.96) times the square root of the estimated variance of the estimator (0.078/4892)^(0.5) to the estimated visit rate (0.078). The procedure for constructing the confidence intervals for difference in webpage visits per invitation (treatment minus control) replaces the variance of the estimator from the treatment (e.g. 0.078/4892 for climate change treatment) with the sum of the variance from the two treatments being compared (e.g. 0.078/4892 + 0.081/4892 for comparing climate treatment to climate control).

### Oversight

This study does not meet the definition of human subjects research because there was no personal identifiable information in the evaluation. Therefore, the University of Delaware Institutional Review Board (IRB) determined prior to the intervention that this study, under the working title “[880101–1] Histosols (Organic Soils) Conservation Decisions by Farmers,” was exempt from IRB review according to federal regulations. A request for Waiver of Consent was granted under 45 CFR 46.117 (c)(2).

## Results and discussion

**Fig 1:**
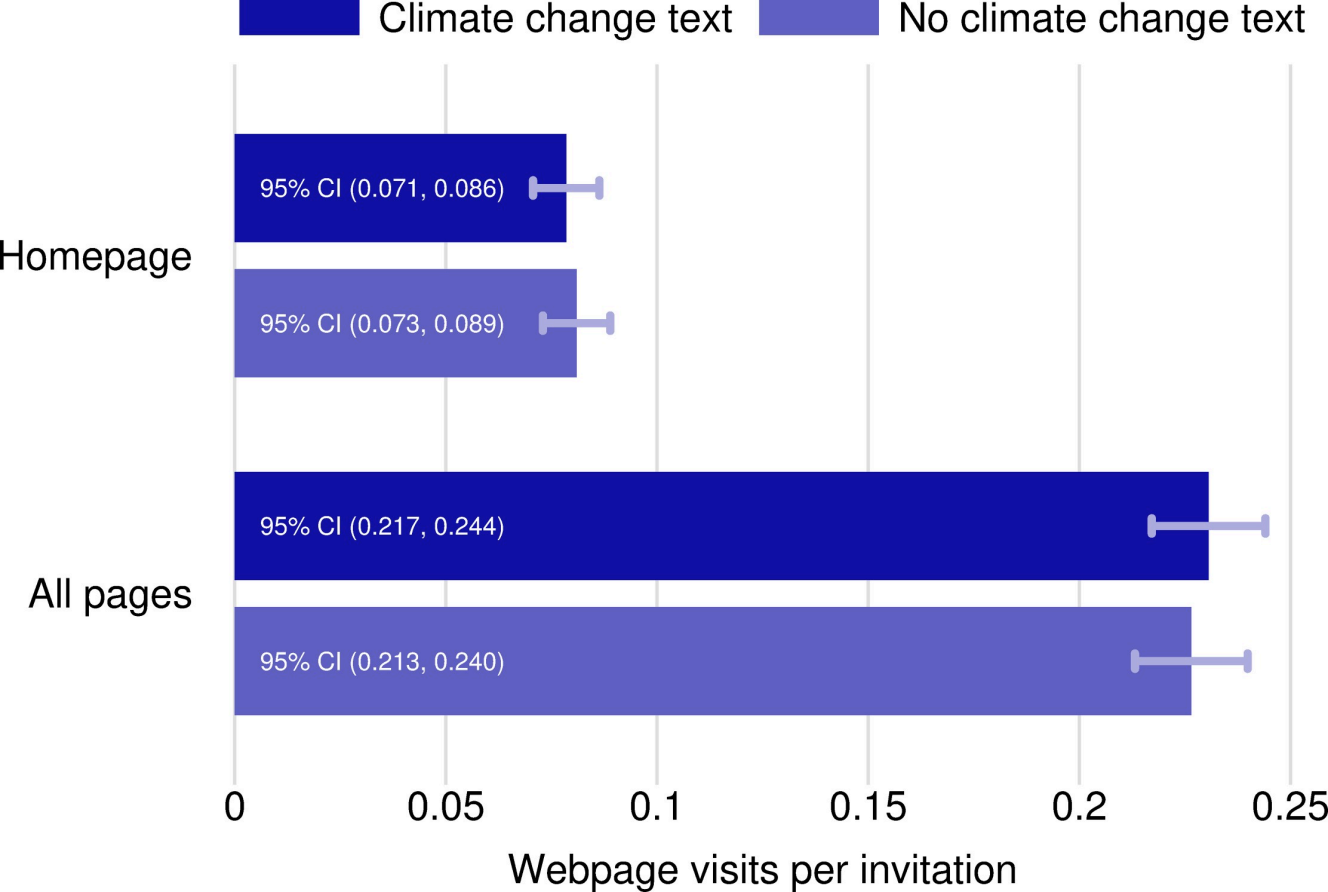
Webpage visits by climate treatment. Webpage visit rates are constructed by dividing the total number of visits to the specified pages by the number of producers sent mailings in that treatment arm. To construct confidence intervals, it is assumed that the number of website visits for a particular invitation is an independent draw from a Poisson distribution, which has the characteristic that the mean is equal to the variance. We apply critical values from the t-distribution when constructing confidence intervals because, under the assumption of independent draws, the Central Limit Theorem applies to the sample mean visit rate. The same procedure is used for constructing all the confidence intervals related to website visits per invitation. To illustrate, we describe an example calculation: The upper bound for the 95% confidence interval for homepage visits per climate change invitation is constructed by adding the two-tailed critical value (1.96) times the square root of the estimated variance of the estimator (0.078/4892)^(0.5) to the estimated visit rate (0.078). The procedure for constructing the confidence intervals for difference in webpage visits per invitation (treatment minus control) replaces the variance of the estimator from the treatment (e.g. 0.078/4892 for climate change treatment) with the sum of the variance from the two treatments being compared (e.g. 0.078/4892 + 0.081/4892 for comparing climate treatment to climate control).

**Fig 2:**
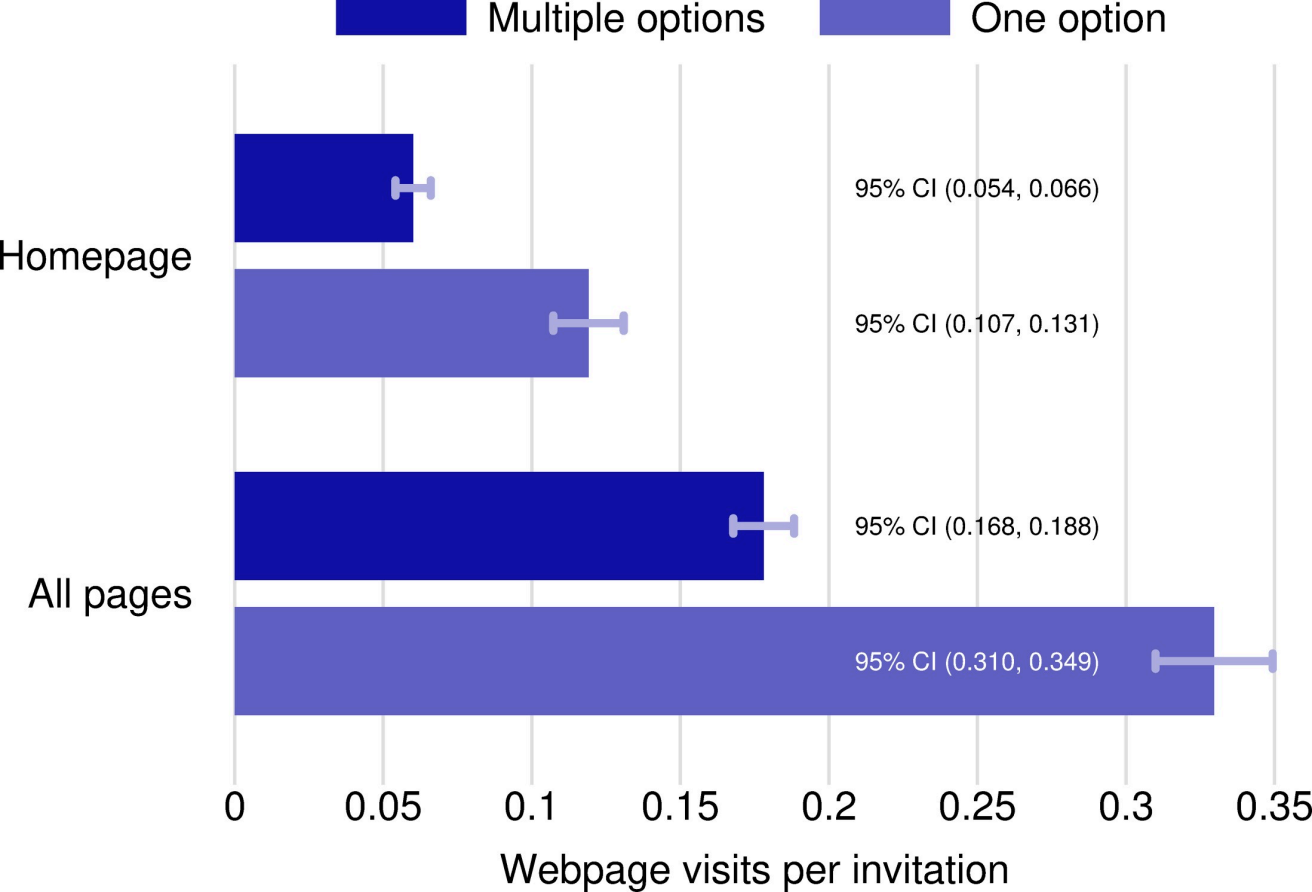
Webpage visits by option treatment. See Fig 1 legend.

**Fig 3:**
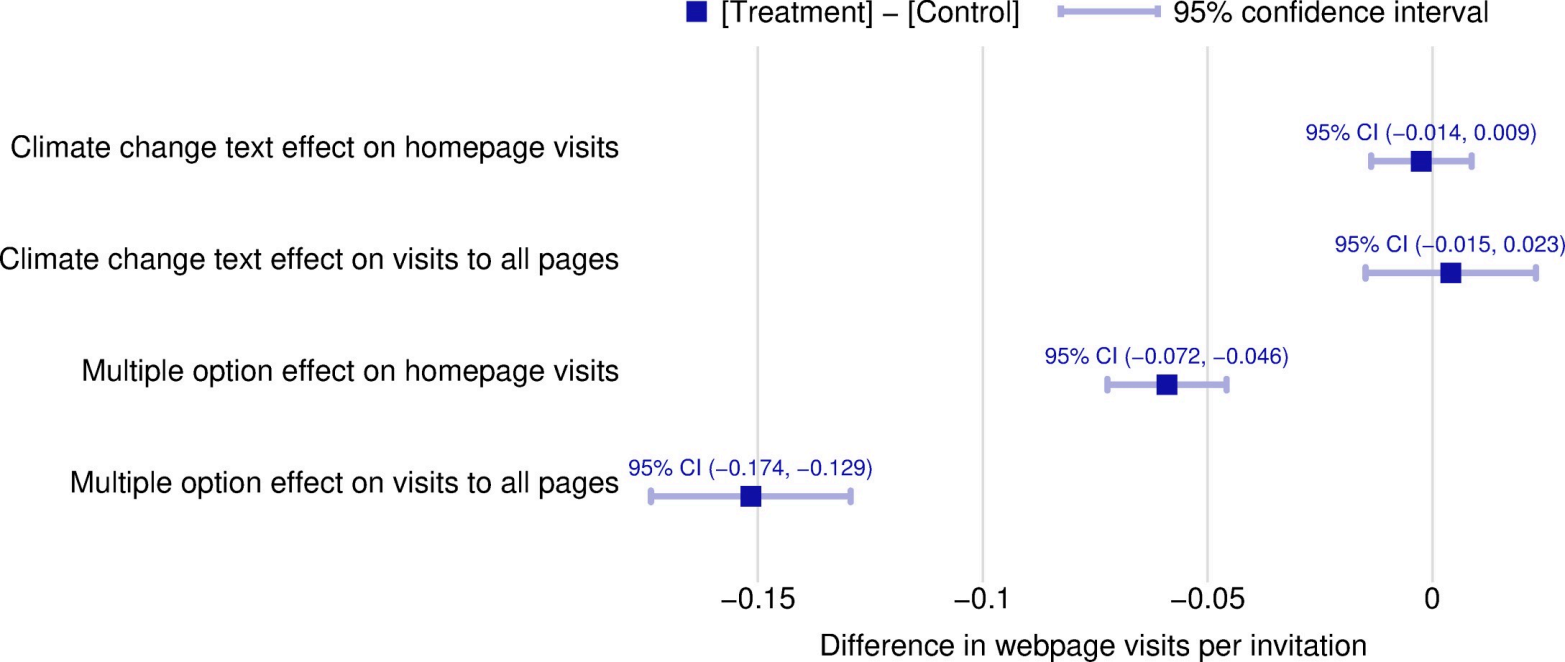
Treatment effects. See Fig 1 legend.

Figs 1 and 2 presents the behavioral effects from the two treatments: the climate change text and the offer of multiple options for action (source data in S1 Table). There is no evidence that explicit text about greenhouse gas emissions from farming and their role in climate change had a negative effect on producer willingness to seek more information about soil conservation practices. The treatment and control websites, which had different web addresses to allow for tracking but identical content, were visited at nearly identical rates (Fig 1), around 0.1 homepage visits per invitation. Using a two-tailed t-test, we fail to reject the null hypothesis of no difference in the webpage visitation rates between the climate treatment group and the climate control group at the 5% significance level (Fig 3).

In contrast, the evidence is inconsistent with classical economic models of behavior. Agricultural producers who were given multiple options for action were about half as likely to visit the website as producers who were given only a single option for action (Fig 2). Using a two-tailed t-test, we reject the null hypothesis of no difference in the webpage visitation rates between the multiple-option treatment group and the single-option control group at the 5% significance level (Fig 3). Most website page visits did not correspond to registration for the webinar; there were 392 hits on the home pages for the webinar treatment websites, and there were 43 webinar registrants. Offering two options for action thus had a negative impact on the likelihood that producers took any action compared to offering one option.

Our results challenge two aspects of conventional wisdom about conservation outreach. First, despite the common belief that mentioning climate change undermines outreach to U.S. agricultural producers, we found no evidence of a negative effect. Second, we found evidence that offering a second option of attending a webinar discouraged producers from visiting an informational website.

We offer a few notes of caution in interpreting our results. First, our measure of behavior change is located early on the causal path to conservation outcomes. Learning about conservation practices is a prerequisite for implementing those practices, but ultimately the outcomes we care most about are downstream from these initial steps. In the context of this experiment, it is even possible that the lower website visitation rates among producers in the webinar treatment were present alongside deeper levels of engagement among the producers who did choose to attend the webinar. If website visitation rates do not correspond closely to engagement, then that outcome might not be a good indicator for judging the downstream impact of the treatment on implementing conservation practices. Similarly, it was not possible to measure whether the depth of engagement on the website varied by climate treatment. We can tell that the website visitation rates in the climate treatment group and the climate control group were not statistically different, but it is possible that there were deeper levels of engagement in one group than the other.

Second, we cannot be sure what producers reacted to in the outreach letter and reminders. Recipients may have read the text about the website and the webinar, but not the italicized and bolded text about the private and public benefits of the conservation practices. In other words, it is possible that the encouraged action had high salience, but the motivation for the action had low salience. Because the experiment was embedded in USDA operations, producers in the sample could not easily be surveyed after the outreach was done to learn more about their motivations related to the observed behavior.

Third, it is possible that climate skepticism is correlated with responsiveness to any outreach effort, such that mentioning climate change does discourage some producers from taking action, but these same producers are also discouraged from taking action in response to any outreach campaign, whether it mentions climate change or not. To shed light on our results, complementary qualitative research that explores the observed quantitative patterns is important.

### Summary and conclusion

As part of outreach to promote soil conservation practices, the USDA used a randomized controlled trial to test two commonly held beliefs about conservation outreach. First, although it was conventional wisdom at USDA that acknowledging the link between climate change and agricultural production would discourage conservation action, we found no evidence of a detrimental impact of acknowledging that link. Second, although it was conventional wisdom at USDA that offering a menu of actions is weakly better than encouraging a single action, we found that agricultural producers were less likely to take steps towards action by visiting a website when offered an additional option of attending a webinar. One important caveat to these results is that our outcome measure, website visitation rates, is an early step on the pathway to adopting conservation practices. We focus on this intermediate outcome because it is what we have access to, but ideally, we would want to examine the impact of the treatment on subsequent conservation practices.

We believe our experimental results show how conservation could benefit from applying the same scientific lens to questions about human behavior change that we apply to other questions about conservation, such as the measurement of the environmental benefits of different conservation practices. We hope our results will spur new scholar-practitioner collaborations that embed experimental designs into real-world agricultural outreach programs. The cumulative evidence can help identify the most effective ways to conduct outreach to agricultural producers in the US and elsewhere that, ultimately, motivate behaviors that promote adoption of conservation practices.

## Supporting information

S1 Fig Treatment letters. A) No climate change reference, no webinar. B) Climate change reference, no webinar. C) No climate change reference, webinar. D) Climate change reference, webinar.
(TIF)

S2 Fig First reminder postcards. A) No climate change reference, no webinar, front. B) No climate change reference, no webinar, back. C) Climate change reference, no webinar, front. D) Climate change reference, no webinar, back. E) No climate change reference, webinar, front. F) No climate change reference, webinar, back. G) Climate change reference, webinar, front. H) Climate change reference, webinar, back.
(TIF)

S3 Fig Second reminder postcards. A) No climate change reference, no webinar, front. B) No climate change reference, no webinar, back. C) Climate change reference, no webinar, front. D) Climate change reference, no webinar, back. E) No climate change reference, webinar, front. F) No climate change reference, webinar, back. G) Climate change reference, webinar, front. H) Climate change reference, webinar, back.
(TIF)

S1 Table Page hit counts. These are the page hit counts that underlie the estimates reported in the main text.
(DOCX)
