# Supplementary figures and images for: Conservation outreach that acknowledges human contributions to climate change does not inhibit action by U.S. farmers: Evidence from a large randomized controlled trial embedded in a federal program on soil health
Source: PLoS One. 2021 Jul 1;16(7):e0253872. doi: 10.1371/journal.pone.0253872 (PMC8248691; doi:10.1371/journal.pone.0253872)

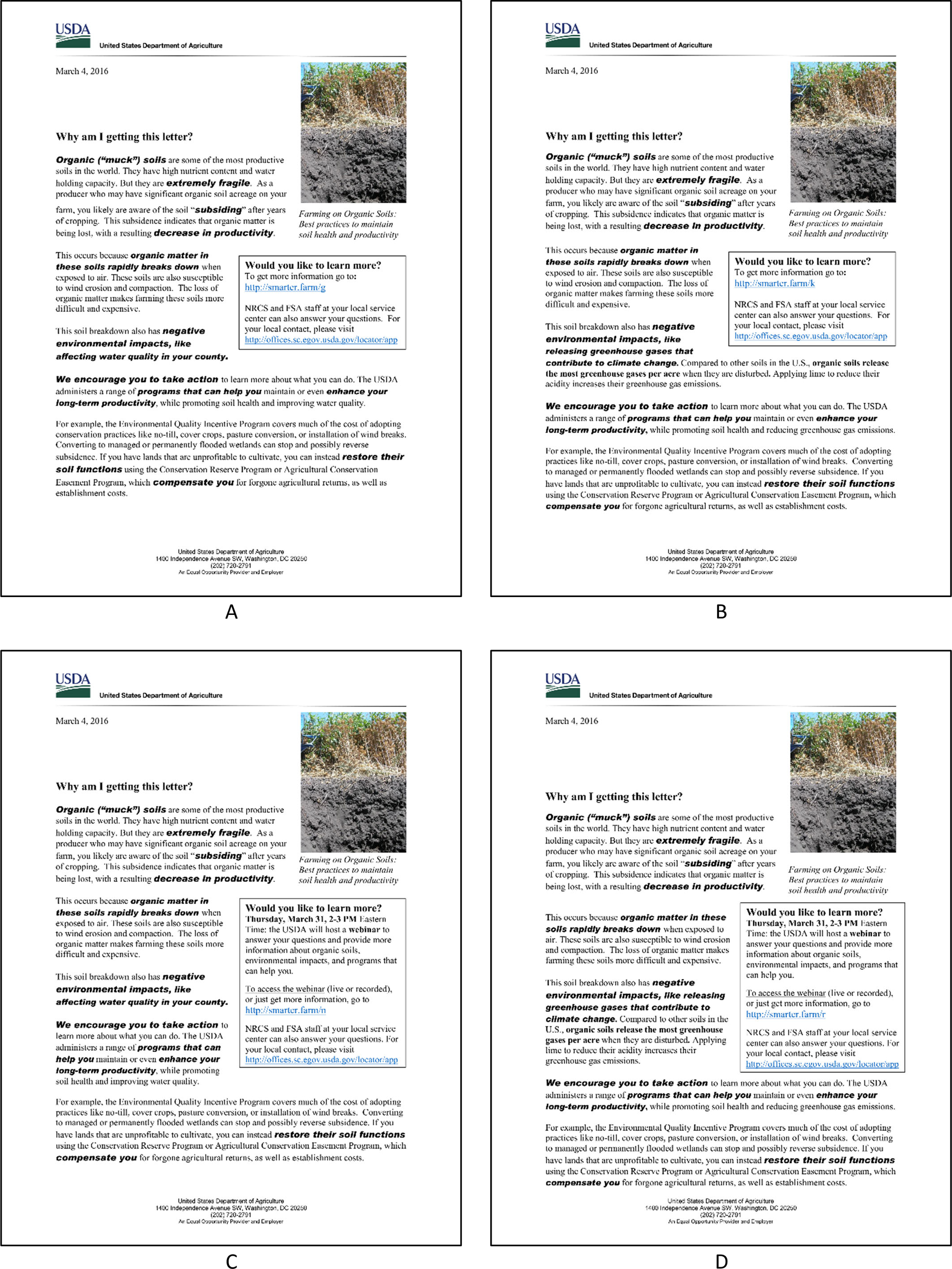

Supplement: S1 Fig — A) No climate change reference, no webinar. B) Climate change reference, no webinar. C) No climate change reference, webinar. D) Climate change reference, webinar. (TIF) [file pone.0253872.s001.tif]

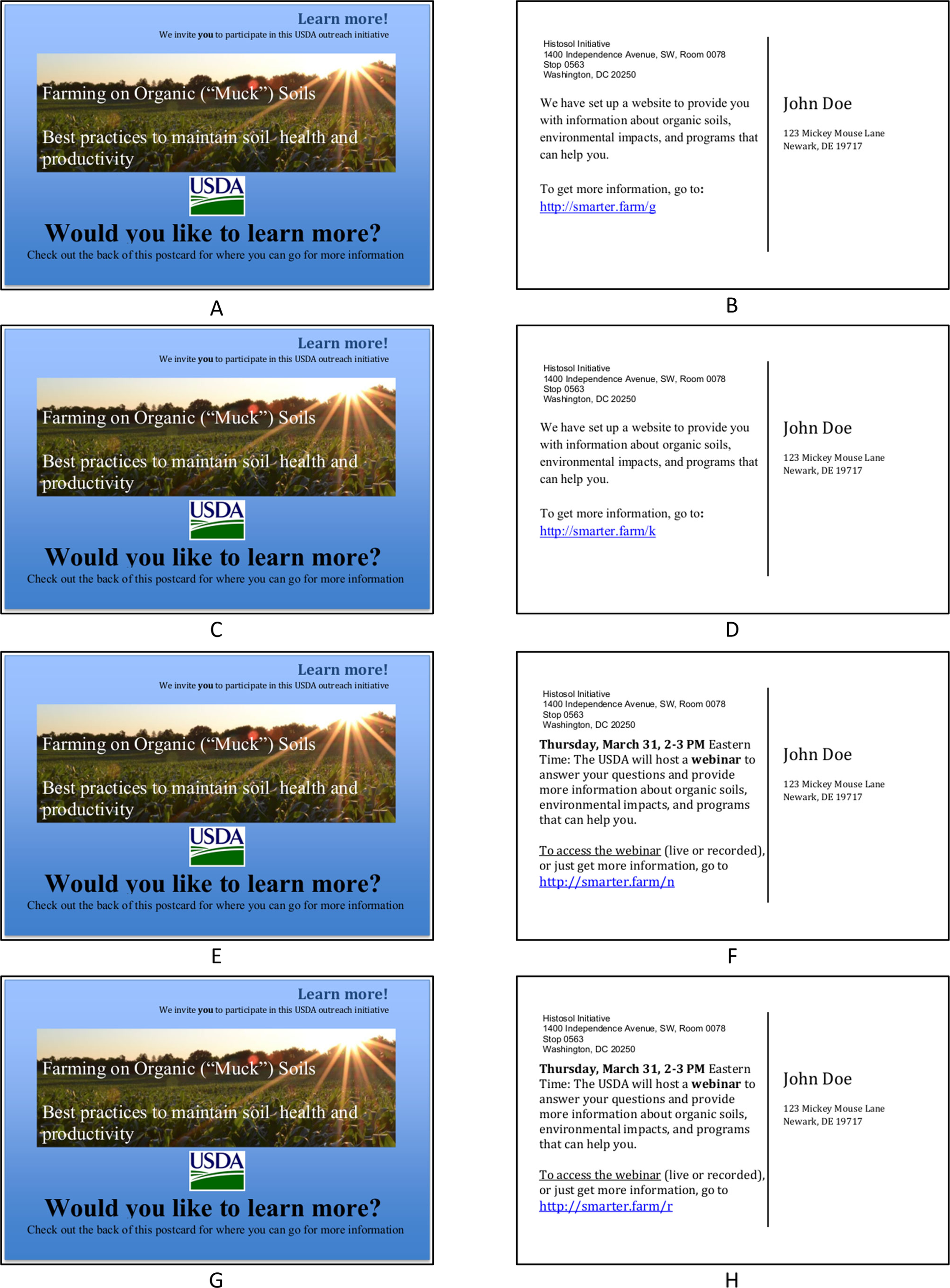

Supplement: S2 Fig — A) No climate change reference, no webinar, front. B) No climate change reference, no webinar, back. C) Climate change reference, no webinar, front. D) Climate change reference, no webinar, back. E) No climate change reference, webinar, front. F) No climate change reference, webinar, back. G) Climate change reference, webinar, front. H) Climate change reference, webinar, back. (TIF) [file pone.0253872.s002.tif]

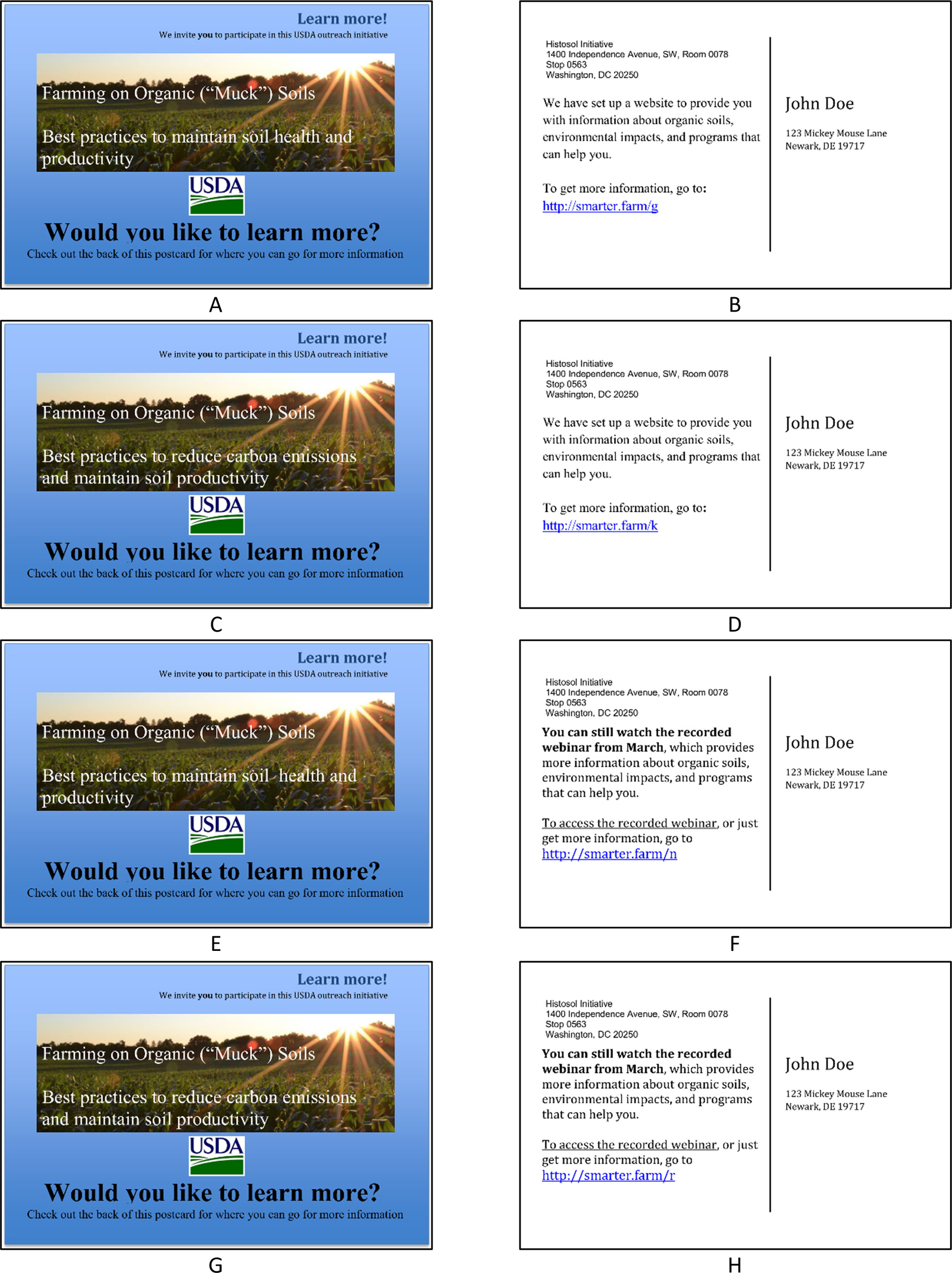

Supplement: S3 Fig — A) No climate change reference, no webinar, front. B) No climate change reference, no webinar, back. C) Climate change reference, no webinar, front. D) Climate change reference, no webinar, back. E) No climate change reference, webinar, front. F) No climate change reference, webinar, back. G) Climate change reference, webinar, front. H) Climate change reference, webinar, back. (TIF) [file pone.0253872.s003.tif]
