# Supplementary material for: Conservation outreach that acknowledges human contributions to climate change does not inhibit action by U.S. farmers: Evidence from a large randomized controlled trial embedded in a federal program on soil health
Source: PLoS One. 2021 Jul 1;16(7):e0253872. doi: 10.1371/journal.pone.0253872 (PMC8248691; doi:10.1371/journal.pone.0253872)
Supplement: S1 Table — These are the page hit counts that underlie the estimates reported in the main text. (DOCX) [file pone.0253872.s004.docx]

|  | **Homepage** | **All pages** |
| --- | --- | --- |
| **No climate change reference, no webinar** | 200 | 536 |
| **Climate change reference, no webinar** | 188 | 538 |
| **No climate change reference, webinar** | 196 | 572 |
| **Climate change reference, webinar** | 196 | 590 |
